# Trends in Heart Failure Hospitalizations in the Abruzzo Region, Italy, 2018–2023: Implications for Preventive Care and Integrated Primary Care Pathways

**DOI:** 10.3390/healthcare14162567

**Published:** 2026-08-17

**Authors:** Piera Scampoli, Giuseppe Di Martino, Francesca Meo, Pamela Di Giovanni, Francesco Cipollone, Donatella Taddeo, Edoardo Trebbi, Ferdinando Romano, Tommaso Staniscia

**Affiliations:** 1 Unit of Hygiene, Epidemiology and Public Health, Local Health Authority of Lanciano-Vasto-Chieti, 66100 Chieti, Italy; piera.scampoli@asl2abruzzo.it (P.S.); tommaso.staniscia@unich.it (T.S.); 2 Department of Medicine and Ageing Sciences, “G. d’Annunzio” University of Chieti-Pescara, 66100 Chieti, Italy; pamela.digiovanni@unich.it; 3 Unit of Epidemiology and Health Statistics, Local Health Authority of Pescara, 65100 Pescara, Italy; 4 District Operational Unit 3, Local Health Authority of Lanciano-Vasto-Chieti, 66100 Chieti, Italy; francesca.meo@asl2abruzzo.it; 5 School of Public Health, “G. d’Annunzio” University of Chieti-Pescara, 66100 Chieti, Italy; francesco.cipollone@studenti.unich.it (F.C.); donatella.taddeo@studenti.unich.it (D.T.); 6 Department of Public Health and Infectious Diseases, “La Sapienza” University of Rome, 00100 Rome, Italy; edoardo.trebbi@uniroma1.it (E.T.); ferdinando.romano@uniroma1.it (F.R.)

**Keywords:** heart failure, epidemiology, primary care, preventable hospitalizations, Italy

## Abstract

**Background**: Hospitalizations for heart failure are commonly considered an indirect indicator of outpatient care effectiveness and continuity between hospital and community services. In 2021, the Abruzzo Region introduced a Diagnostic-Therapeutic Care Pathway (PDTA) for chronic heart failure, aimed at improving integrated management and reducing avoidable exacerbations. This study analysed trends in heart failure hospitalizations among residents of the Abruzzo Region from 2018 to 2023. **Methods**: We conducted a retrospective population-based study using regional hospital discharge records. Hospitalizations were identified according to the ICD-9-CM codes. Only admissions among adults with heart failure recorded as the principal diagnosis were included. Annual crude and age- and sex-standardized hospitalization rates were calculated using the 2018 Abruzzo resident population as the standard population. Temporal trends were assessed using Joinpoint regression. **Results**: Overall, 27,247 hospitalizations for heart failure were included. Annual admissions decreased from 5654 in 2018 to 3791 in 2023, corresponding to an approximate 33% reduction. Standardized hospitalization rates declined from 4.38 per 1000 inhabitants in 2018 to 2.85 per 1000 in 2023. Joinpoint analysis showed a significant decreasing trend, with an average annual percentage change of −9.1% (95% CI: −15.3 to −2.5). **Conclusions**: Heart failure hospitalizations significantly decreased in Abruzzo between 2018 and 2023. These findings may reflect changes in hospital use, pandemic-related effects, and progressive improvements in territorial chronic care management.

## 1. Introduction

Heart failure is a major challenge for contemporary health systems. Its clinical and epidemiological relevance is closely linked to population ageing, the increasing burden of chronic diseases and multimorbidity, and the need for long-term, coordinated care. Although therapeutic advances have improved the prognosis of many patients, heart failure continues to be associated with frequent exacerbations, repeated hospital admissions, poor quality of life and high mortality [1,2]. For these reasons, it remains one of the chronic conditions with the greatest impact on hospital activity and healthcare expenditure.

Hospitalization represents a crucial event in the natural history of heart failure. It often marks a phase of clinical instability, exposes patients—particularly older and frail individuals—to functional decline and adverse outcomes, and is frequently followed by early readmission. At the same time, hospitalization for heart failure should not be interpreted only as an acute clinical episode. In many cases, it reflects the effectiveness of the broader care pathway preceding admission, including primary care, outpatient cardiology, patient education, treatment adherence, timely recognition of worsening symptoms and continuity after previous hospital discharge. For this reason, heart failure admissions are widely used in health-services research as indicators of potentially avoidable hospitalizations and ambulatory care sensitive conditions [3,4].

This interpretation is particularly relevant for health systems aiming to shift the management of chronic diseases from hospital-centred models to proactive, community-based care. Contemporary guidelines emphasize that heart failure requires a structured and multidisciplinary approach, including early diagnosis, risk stratification, optimization of evidence-based therapies, management of comorbidities, self-care support and planned follow-up after discharge [5,6,7]. Evidence from disease management and transitional care programs suggests that integrated interventions can reduce readmissions and improve continuity of care, especially when they include close follow-up, medication reconciliation, patient education and coordination between hospital and territorial services [8,9,10].

In Italy, the reorganization of chronic care has increasingly relied on the development of Diagnostic-Therapeutic Care Pathways, known as “Percorsi Diagnostico-Terapeutico Assistenziali” (PDTA). These pathways are intended to translate clinical guidelines into local organizational models, defining the roles of professionals, referral criteria, follow-up schedules and interactions between hospital and community services. For chronic heart failure, a PDTA may therefore represent not only a clinical tool, but also a public health strategy aimed at improving appropriateness, preventing avoidable exacerbations and reducing unnecessary hospital use.

The Abruzzo Region introduced a specific PDTA for chronic heart failure in 2021, through Regional Government Decree No. 524 of 13 August 2021 [11,12]. The regional PDTA was designed to define a structured pathway for patients with chronic heart failure across different stages of disease. Its main components included the identification of roles and responsibilities among general practitioners, cardiologists, hospital units, district services, rehabilitation facilities and home care; the definition of referral criteria between primary care, outpatient cardiology and hospital settings; indications for hospitalization in cases of acute decompensation; structured follow-up after discharge; and the possibility of using telemedicine or teleconsultation in selected clinical situations. The pathway was therefore intended to improve continuity of care, standardize clinical management and support earlier recognition of clinical instability.

Abruzzo offers a particularly interesting setting for the evaluation of these issues. The region is characterized by demographic ageing, heterogeneous geographical areas, inland municipalities and differences in healthcare accessibility across Local Health Authorities. In such a context, hospitalization rates for heart failure may provide useful information not only on disease burden, but also on the ability of the healthcare system to provide timely and continuous care close to patients’ homes.

This study aimed to analyze the trend of hospitalizations for heart failure among residents of the Abruzzo Region from 2018 to 2023. Despite the relevance of heart failure as an ambulatory care sensitive condition, recent population-based evidence on hospitalization trends in Abruzzo is limited, particularly for the period encompassing the COVID-19 pandemic and the introduction of the regional PDTA for chronic heart failure. This study does not aim to evaluate the causal effectiveness of the PDTA. Rather, it provides a descriptive assessment of temporal trends in heart failure admissions before, during and after major healthcare system changes, offering information useful for regional planning and for the generation of future evaluative hypotheses.

## 2. Materials and Methods

### 2.1. Study Design and Data Source

This retrospective population-based study was conducted using hospital discharge records (HDR) from the Abruzzo Region, Italy, for the period 2018–2023. HDR are routinely collected administrative data and represent a consolidated source for analysing healthcare utilization, hospitalization patterns and outcomes related to chronic diseases. In the field of heart failure, these data are particularly useful for monitoring hospital burden and for evaluating indicators related to potentially avoidable admissions.

Hospitalizations for heart failure were identified using the criteria adopted by the Italian National Agency for Regional Healthcare Services for the indicator “hospitalization for heart failure” included in the Italian National Outcomes Programme [13]. All hospital admissions among individuals older than 18 years were screened for ICD-9-CM diagnosis codes identifying heart failure.

The following ICD-9-CM codes were used: 398.91, 402.01, 402.11, 402.91, 404.01, 404.03, 404.11, 404.13, 404.91, 404.93, 428.0, 428.1, 428.2X 428.3X, 428.4X and 428.9. These codes identify rheumatic heart failure, hypertensive heart disease with congestive heart failure, hypertensive heart and renal disease with congestive heart failure, congestive heart failure, left-sided heart failure, systolic heart failure, diastolic heart failure, combined systolic and diastolic heart failure and unspecified heart failure.

Only admissions with one of these codes recorded as the principal diagnosis were included. Hospitalizations of non-residents, transferred patients, and admissions involving surgical interventions or other cardiac procedures were excluded, in accordance with the methodology used for the national indicator [13]. The analysis was carried out on an annual basis. The unit of analysis was the hospital admission rather than the individual patient. Therefore, if the same patient was hospitalized more than once during the study period, each admission was counted as a separate event. The outcome should consequently be interpreted as a population-based heart failure admission rate, not as an incidence rate of hospitalized patients.

### 2.2. Statistical Analysis

Continuous variables were reported as mean and standard deviation or as median and interquartile range, accordingly to their distribution. Categorical variables were summarized as frequencies and percentages.

The annual hospitalization rate for heart failure was calculated by using the number of heart failure hospitalizations as the numerator and the resident population of the Abruzzo Region as the denominator. Demographic data by year, age and sex were obtained from the Italian National Institute of Statistics [14].

Crude rates were calculated for each year of observation. To account for changes in the demographic structure of the population and to allow comparisons over time, hospitalization rates were standardized by age and sex using the direct standardization method. The resident population of the Abruzzo Region in 2018, the first year of observation, was used as the standard population.

Temporal trends in standardized hospitalization rates were assessed through Joinpoint regression analysis. Joinpoint Regression Program version 4.6.0.0 was used to estimate the annual percentage change and the average annual percentage change over the entire study period [15]. The final model was selected as the best-fitting model for the observed data and consisted of one or more linear segments connected by joinpoints.

All statistical tests were two-sided. A *p*-value below 0.05 was considered statistically significant. Statistical analyses were performed using Stata version 20 (Stata Corp LLC, College Station, TX, USA) [16].

## 3. Results

Between 2018 and 2023, 985,255 hospital admissions were recorded in the Abruzzo Region. Among these, 67,877 admissions had at least one diagnosis of heart failure, corresponding to 6.89% of all hospitalizations. When the analysis was restricted to admissions with heart failure recorded as the principal diagnosis, 30,435 hospitalizations were identified, accounting for 3.09% of all admissions. After excluding admissions involving surgical interventions or other cardiac procedures, the final study population included 27,247 hospitalizations for heart failure, corresponding to 2.77% of all hospital admissions during the study period (Table 1).

The annual number of heart failure hospitalizations decreased from 5654 in 2018 to 3791 in 2023, corresponding to an overall reduction of approximately 33%. The proportion of heart failure admissions among all hospitalizations also decreased, from 3.46% in 2018 to 2.27% in 2023.

**Figure 1:**
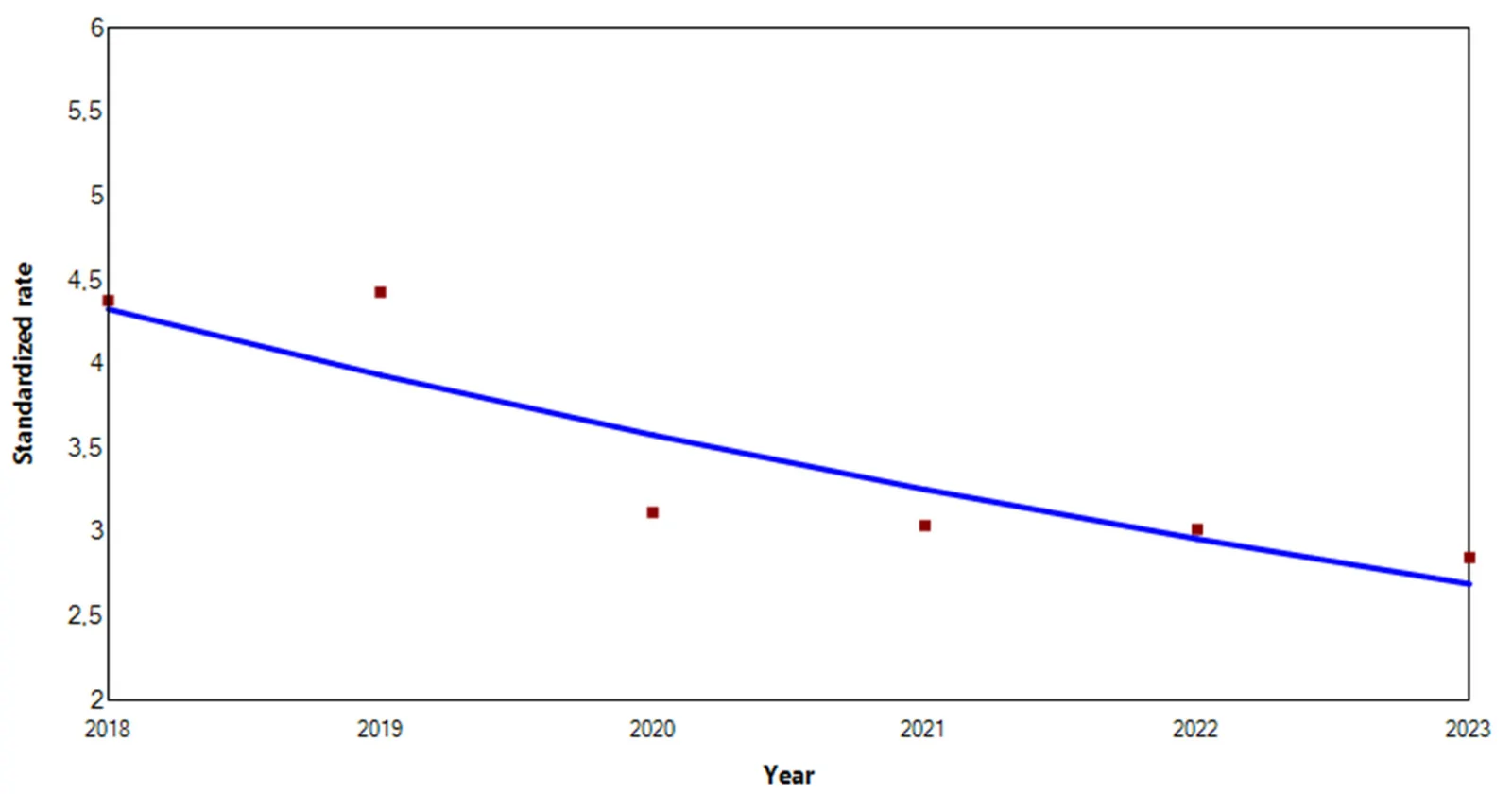
Age- and gender-standardized trend in HF hospitalizations. (Dots represent the yearly standardized rate and the blue line represents the fitted regression model).

For descriptive purposes, the study period may be divided into three phases: 2018–2019 as the pre-COVID and pre-PDTA phase, 2020–2021 as the pandemic and transition phase, and 2022–2023 as the early post-acute pandemic and post-PDTA implementation phase. Standardized hospitalization rates were 4.38 and 4.43 per 1000 inhabitants in 2018 and 2019, respectively; they decreased to 3.12 and 3.04 per 1000 inhabitants in 2020 and 2021; and remained below pre-pandemic levels in 2022 and 2023, with rates of 3.02 and 2.85 per 1000 inhabitants, respectively (Figure 1).

Joinpoint regression confirmed a statistically significant downward trend over the entire study period. The average annual percentage change was −9.1% (95% CI: −15.3 to −2.5), indicating a significant reduction in standardized hospitalization rates for heart failure in Abruzzo between 2018 and 2023.

Heart failure hospitalizations were slightly more frequent among men than among women. Overall, 51.77% of admissions occurred in male patients and 48.23% in female patients, with a statistically significant difference between sexes. Men were younger than women at the time of hospitalization, with a mean age of 76.54 years compared with 81.86 years among women. During the study period, the mean age of hospitalized patients decreased from 79.98 years in 2018 to 78.13 years in 2023.

**Figure 2:**
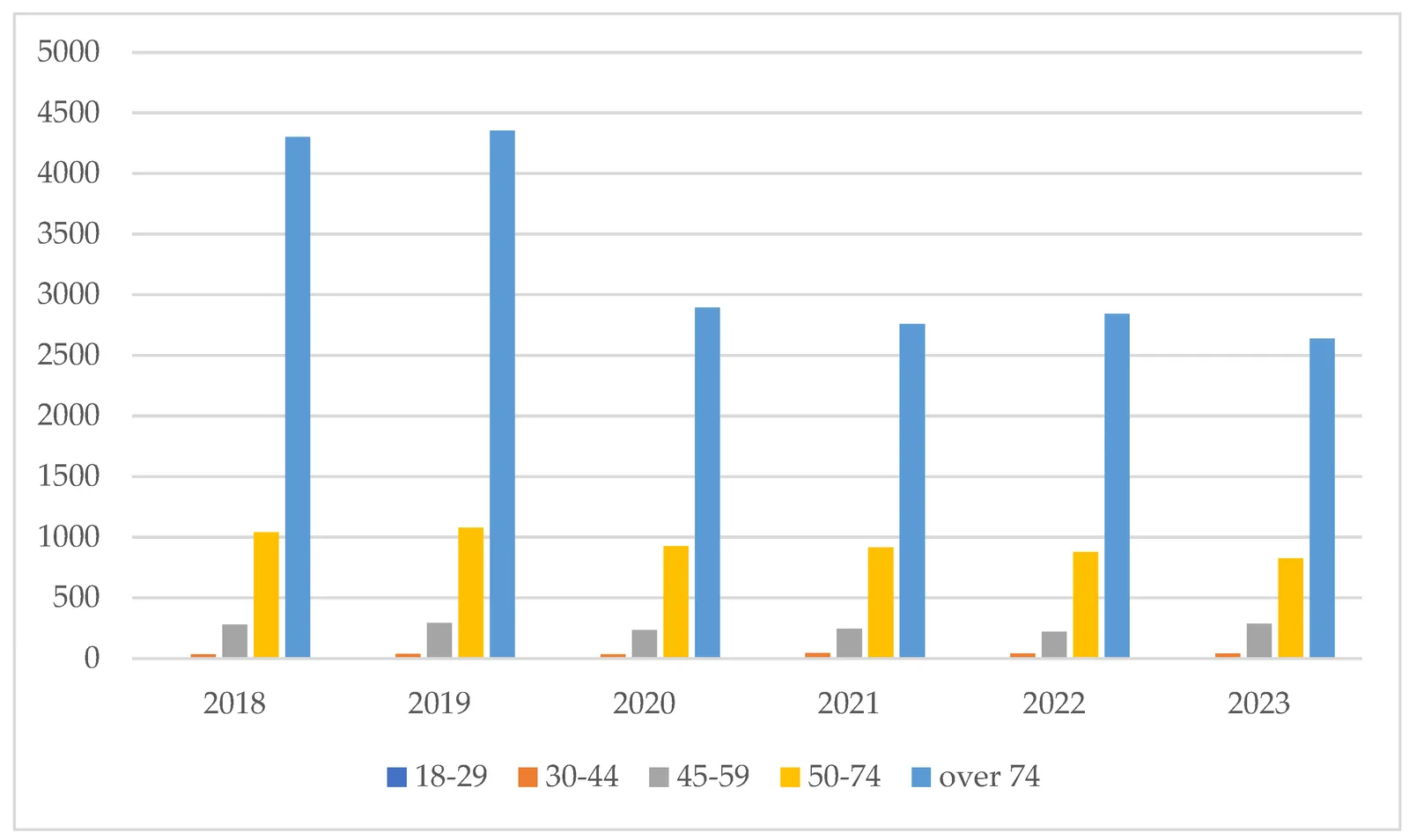
HF hospitalization distribution by age group.

The distribution by age group confirmed the close relationship between heart failure and older age. More than 72% of hospitalizations occurred among patients older than 74 years, while 20.76% involved patients aged 50–74 years. Only 6.63% of admissions occurred among individuals younger than 50 years. The reduction in hospitalizations was mainly driven by the oldest age group: among patients older than 74 years, admissions decreased by 38.68% between 2018 and 2023, whereas trends in younger age groups were substantially stable (Figure 2).

When hospitalizations were analyzed by four Local Health Authorities (Aziende Sanitarie Locali, ASLs), differences emerged across the regional territory. In 2018, the highest hospitalization rate was observed in the ASL of Lanciano-Vasto-Chieti, with 5.85 admissions per 1000 inhabitants, followed by Avezzano-L’Aquila-Sulmona, with 4.23 admissions per 1000 inhabitants.

Over the whole study period, the highest proportion of heart failure admissions among all hospitalizations was observed in Teramo, where heart failure accounted for 3.60% of admissions, followed by Lanciano-Vasto-Chieti, with 3.39%. Lower proportions were observed in Avezzano-L’Aquila-Sulmona and Pescara, with 2.45% and 2.02%, respectively (Table 2).

A decreasing trend was observed in all four ASLs, although with different magnitudes. The most marked decline was recorded in Lanciano-Vasto-Chieti (ASL 2), where the hospitalization rate decreased with an average annual percentage change of −11.5% (95% CI: −18.2 to −5.4). In this area, the reduction was particularly evident between 2018 and 2021, with an annual percentage change of −26.2% (95% CI: −40.8 to −16.3), followed by a non-significant increase between 2021 and 2023.

**Figure 3:**
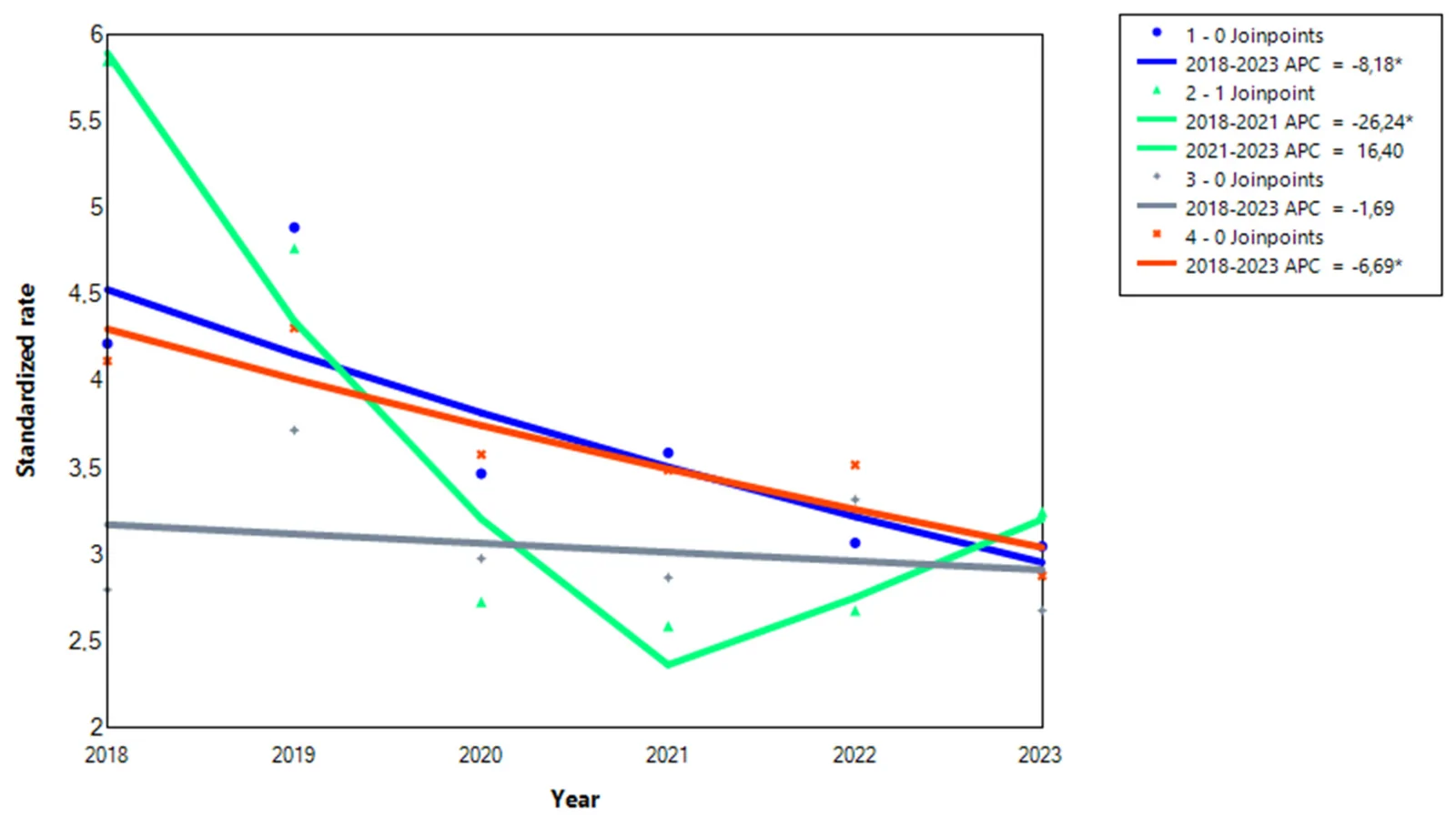
Trends in standardized hospitalization rates for heart failure by Local Health Authority (Azienda Sanitaria Locale, ASLs) in the Abruzzo Region, 2018–2023.

Significant reductions were also observed in Avezzano-L’Aquila-Sulmona (ASL 1) and Teramo (ASL 4), with average annual percentage changes of −8.2% (95% CI: −14.2 to −1.8) and −6.7% (95% CI: −10.6 to −2.6), respectively. In Pescara (ASL 3), the trend was not statistically significant, with an average annual percentage change of −1.7% (95% CI: −10.4 to 7.7), as reported in Figure 3.

In-hospital mortality among patients admitted for heart failure remained high throughout the study period. Overall, 8.23% of heart failure hospitalizations ended in death. However, a decrease was observed over time, from 8.88% in 2018 to 7.57% in 2023 (*p* = 0.024, 95%CI 0.19–2.43). Intermediate values were 8.12% in 2019, 8.19% in 2020, 8.14% in 2021 and 8.22% in 2022.

## 4. Discussion

This study found a significant decrease in hospitalizations for heart failure in the Abruzzo Region between 2018 and 2023. The reduction was evident both in absolute numbers and in age- and sex-standardized rates. Overall, admissions decreased by about one third, and Joinpoint analysis confirmed a significant average annual reduction of 9.1%. The decline was particularly marked among patients older than 74 years, the group in which heart failure is most frequent and in which the consequences of hospitalization are usually more severe.

The interpretation of the observed trend should take into account the different phases covered by the study period. The years 2018–2019 may be considered the pre-COVID and pre-PDTA phase, providing a baseline picture of heart failure hospitalizations before the major organizational changes that occurred subsequently. The years 2020–2021 correspond to the period most affected by the COVID-19 pandemic, during which access to hospital care were substantially disrupted. Finally, the years 2022–2023 represent an early post-acute pandemic and post-PDTA implementation phase. The persistence of hospitalization rates below pre-pandemic levels during these years may suggest a more stable change in the management of patients with heart failure, potentially consistent with improved territorial care. However, this interpretation remains hypothesis-generating and cannot be considered evidence of a direct causal effect.

The findings are relevant because heart failure remains a condition with a high clinical and organizational impact. It is not only a frequent cause of hospital admission, but also a marker of complex healthcare needs. Patients admitted for heart failure are often older, affected by multiple chronic conditions and exposed to a high risk of readmission, disability and death [1,2]. In this sense, the observed reduction in hospitalizations may represent an important signal for regional health planning, especially if interpreted within the broader transition from hospital-centred care to integrated territorial management.

Hospitalizations for heart failure are commonly considered among ambulatory care sensitive conditions. This does not imply that all admissions are avoidable. Many patients require hospital care because of severe decompensation or advanced disease. However, part of the hospital burden may be influenced by the quality and continuity of outpatient care, the timeliness of follow-up, adherence to treatment, patient and caregiver education, and the ability of primary care and specialist services to recognize clinical deterioration at an early stage [3,4]. For this reason, trends in heart failure hospitalization rates may provide an indirect measure of how effectively chronic cardiovascular disease is managed outside hospital.

The reduction observed in Abruzzo is consistent with this framework, although it should be interpreted carefully. The study period included the COVID-19 pandemic, which had a profound effect on access to hospital care and on the organization of outpatient services. The sharp decrease in 2020 may partly reflect changes in health-seeking behaviour, restrictions in access to healthcare, reduced use of hospital services by frail patients, and pressure on acute care facilities. Therefore, the decline between 2019 and 2020 cannot be attributed only to improved territorial management. However, the persistence of lower hospitalization rates in 2021, 2022 and 2023 suggests that the change was not limited to the first pandemic year.

A central element in the interpretation of these results is the introduction of the regional PDTA for chronic heart failure in Abruzzo in 2021 [11,12]. The PDTA was designed to standardize the care pathway, define responsibilities among healthcare professionals and promote continuity between hospital and community services. Its structure reflects the principles of contemporary heart failure guidelines, which recommend multidisciplinary care, planned follow-up, patient education, therapeutic optimization and early identification of worsening symptoms [5,6,7]. It is also coherent with the Chronic Care Model, which emphasizes proactive care, integration among professionals and a more active role of patients in the management of chronic illness [8].

From this perspective, the reduction in hospitalizations after 2021 may be interpreted as a population-level signal potentially consistent with improved chronic care management. The marked reduction among patients older than 74 years should be interpreted cautiously. Although older adults are the group most likely to benefit from structured chronic care pathways, they were also disproportionately affected by the COVID-19 pandemic. Healthcare avoidance, restricted access to hospitals, changes in admission thresholds, increased mortality outside hospital and disruption of outpatient services may all have contributed to the observed decline. Older patients are more likely to be affected by frailty, multimorbidity and polypharmacy, and they often require continuous monitoring and coordinated care. They are also the group most likely to benefit from structured pathways, proactive follow-up and stronger integration between general practitioners, cardiologists, district services and home care [9,10]. Nevertheless, the present study cannot demonstrate a causal effect of the PDTA. Its full implementation likely required time, and the available data did not allow us to measure whether individual patients were actually managed according to the pathway.

The results should also be considered in relation to previous research conducted in Abruzzo using administrative data to assess potentially preventable hospitalizations. Di Martino et al. analysed diabetes-related preventable hospitalizations over an 11-year period in a region of Southern Italy, showing how hospital discharge records can support the monitoring of avoidable hospital use and help identify patient groups requiring stronger preventive interventions [17]. Similarly, Di Giovanni et al. examined paediatric ambulatory care sensitive hospitalizations in Abruzzo and highlighted the usefulness of these indicators for assessing primary care performance and territorial inequalities [18]. The present study extends this line of research to heart failure, a condition that is particularly relevant for the evaluation of chronic care pathways and territorial healthcare organization.

The differences observed across ASL deserve attention. Although all areas showed a reduction in hospitalization rates, the magnitude of the decline varied. Lanciano-Vasto-Chieti showed the most pronounced decrease, while the trend in Pescara was not statistically significant. These differences may reflect several factors, including baseline hospitalization rates, demographic structure, burden of disease, coding practices, hospital admission policies, availability of outpatient cardiology services, strength of primary care networks and local implementation of the PDTA. The finding that areas with higher baseline rates experienced larger reductions may suggest a process of convergence toward regional or national benchmarks, but this hypothesis requires further investigation.

Territorial heterogeneity is particularly relevant in Abruzzo, where coastal areas, urban centers, rural municipalities and inland areas coexist within the same regional health system. Accessibility to healthcare services may differ substantially across these settings. In this regard, the study by Trebbi et al. on the optimization of territorial healthcare networks in the Province of L’Aquila provides an important methodological and policy perspective [19]. By applying a capacity-constrained hub-and-spoke allocation algorithm, the authors showed how quantitative models may support the planning of territorial healthcare networks by considering population distribution, service capacity and geographical accessibility. This approach is highly relevant for chronic conditions such as heart failure, in which proximity, continuity and timely access to outpatient care may help prevent decompensation and reduce unnecessary hospital use.

The decrease in in-hospital mortality from 8.88% in 2018 to 7.57% in 2023 is another relevant finding. Mortality among patients admitted for heart failure remained high, confirming the severity of the condition and the vulnerability of hospitalized patients. The observed reduction may reflect improvements in acute care, changes in case mix, earlier management of decompensation or better disease control before hospitalization. However, this result should be interpreted with caution. In-hospital mortality does not capture deaths occurring after discharge, nor does it provide information on 30-day mortality, long-term survival, readmissions or functional outcomes. These indicators are essential to evaluate the real effectiveness of care pathways for heart failure.

The findings have important implications for health policy. Heart failure is a paradigmatic condition for the reorganization of chronic care. Its management requires a model in which hospital care is reserved for acute and complex phases, while stable and post-acute patients are followed through primary care, outpatient cardiology, district services, rehabilitation and home care. The Italian reform of territorial healthcare, formalized by Ministerial Decree No. 77/2022, moves in this direction by promoting Community Health Houses, Community Hospitals, Territorial Operations Centres, family and community nurses, and stronger integration between primary care and specialist care [20]. In a region such as Abruzzo, these instruments may be particularly useful to reduce geographical barriers and improve continuity for frail patients living in inland or rural areas.

The PDTA for chronic heart failure should therefore be viewed as part of a broader reorganization of territorial care. Its effectiveness depends on its translation into routine practice. Key elements include early identification of patients at risk, structured follow-up after discharge, clear referral criteria, systematic medication review, titration of guideline-directed therapies, patient and caregiver education, monitoring of adherence, management of comorbidities, and integration with home care and rehabilitation services [5,6,7,9,10]. Telemedicine may also contribute to strengthening this model, particularly in areas where distance and accessibility represent barriers to care [12,20]. Changes in heart failure pharmacological management during the study period may also have influenced hospitalization and mortality trends. In recent years, guideline-directed medical therapy for heart failure has evolved substantially, with increasing use of angiotensin receptor–neprilysin inhibitors and sodium-glucose cotransporter 2 inhibitors, both of which have been associated with reductions in heart failure hospitalizations and adverse cardiovascular outcomes [21,22]. The progressive implementation of these therapies in clinical practice may have contributed to improved disease stability and lower hospitalization risk. However, the present study did not include pharmaceutical data, information on treatment uptake, adherence, titration or ejection fraction phenotype. Therefore, the potential contribution of changes in pharmacological therapy cannot be quantified and remains an important unmeasured factor.

This study has some limitations. First, the analysis was based on hospital discharge records, which are administrative data collected primarily for management and reimbursement purposes. Although they are useful for population-level monitoring, they may be affected by coding variability and changes in admission practices. Second, heart failure was identified only when recorded as the principal diagnosis. This approach is consistent with the methodology of the national indicator, but it may underestimate the broader burden of heart failure-related hospital care [13]. Third, the study did not include clinical information such as ejection fraction, NYHA class, severity of disease, comorbidities, pharmacological treatment or frailty. Fourth, no information was available on outpatient follow-up, adherence to therapy, primary care management, use of telemedicine, home care or rehabilitation. Finally, the absence of a control group, comparator region or comparator condition represents an important limitation. The marked decline observed in 2020 occurred before the implementation of the PDTA and coincided with the COVID-19 pandemic, which substantially affected access to care, hospital admission patterns and health-seeking behavior. In addition, a relevant limitation is the absence of process measures describing the actual implementation of the PDTA. The hospital discharge records used in this study did not provide information on patient enrolment in the pathway, fidelity to scheduled follow-up and general practitioner involvement. So, the study cannot evaluate implementation fidelity or determine whether individual patients were actually managed according to the PDTA Therefore, the observed reduction cannot be attributed to the PDTA, and the results should be interpreted as descriptive temporal trends.

Despite these limitations, the study provides a regional assessment of heart failure hospitalization trends over a period characterized by both pandemic disruption and relevant organizational changes. The use of standardized rates and Joinpoint regression strengthen the analysis of temporal trends, while stratification by age, sex and Local Health Authority offers useful information for regional planning. Moreover, the study contributes to a growing body of evidence from Abruzzo showing how administrative healthcare data can be used to monitor preventable hospitalizations, evaluate territorial care and support the design of integrated healthcare networks [17,18,19].

## 5. Conclusions

In conclusion, hospital admissions for heart failure decreased in the Abruzzo Region between 2018 and 2023, with the largest decline occurring in 2020 and with rates remaining below pre-pandemic levels in subsequent years. The study describes population-based temporal trends but does not allow causal inference regarding the impact of the regional PDTA introduced in 2021. The findings should be interpreted considering pandemic-related changes in hospital access, evolving heart failure therapies and the absence of individual-level process indicators. Future studies integrating hospital discharge records with outpatient, pharmaceutical, primary care, home care and mortality data are needed to evaluate care pathway implementation, readmissions, treatment patterns, long-term outcomes and equity of access.

## Figures and Tables

**Table 1:** Heart failure hospitalizations and total hospital admissions by year in the Abruzzo Region, 2018–2023.

Year	Heart Failure Hospitalizations	Percentage of Total Admissions (%)
2018	5654	3.46
2019	5765	3.18
2020	4091	2.75
2021	3966	2.47
2022	3980	2.42
2023	3791	2.27
Total	27,247	2.77

**Table 2:** Heart failure hospitalizations and proportion of total admissions by Local Health Authority.

Local Health Authority (ASL)	Heart Failure Hospitalizations	Percentage of Total Admissions (%)
ASL 1—Avezzano-L’Aquila-Sulmona	6576	2.45
ASL 2—Lanciano-Vasto-Chieti	8340	3.39
ASL 3—Pescara	5827	2.02
ASL 4—Teramo	6677	3.60

## Data Availability

Data is not available due to the restriction policy of the Abruzzo region.
